# Migraine and Medical Ramifications: A Comprehensive Overview Based on Observational Study Meta-Analyses

**DOI:** 10.3389/fneur.2021.778062

**Published:** 2021-12-24

**Authors:** Weiwei Chen, Wenqi Qian, Lixian Zhong, Gongwei Jing

**Affiliations:** ^1^Department of Gastroenterology, The First People's Hospital of Zunyi (The Third Affiliated Hospital of Zunyi Medical University), Zunyi, China; ^2^Department of Pharmacy, People's Hospital of Qiandongnan and Dong Autonomous Prefecture, Kaili, China; ^3^Department of Gastroenterology, The First Affiliated Hospital, Jinan University, Guangzhou, China; ^4^Department of Nuclear Medicine, The First People's Hospital of Zunyi (The Third Affiliated Hospital of Zunyi Medical University), Zunyi, China

**Keywords:** migraine, health, medical ramifications, umbrella review, meta-analysis

## Abstract

**Purpose:** An umbrella review was conducted for comprehensively evaluating previous review-based literature together with meta-analysis of observational investigations probing correlations between migraine and medical end-point ramifications in patients. The breadth and validity of these associations were assessed.

**Methods:** Multiple online scientific repositories (including PubMed, Medline, Embase, and Web of Science) were investigated (inception-August 2021) for related meta-analyses focusing on links between migraine and all possible health/medical ramification end-points. A summary effect size and 95% CIs were determined for each identified study with such links. Heterogeneity and small-study influence traces were also evaluated. The AMSTAR 2 platform was employed for evaluating standards of methodology, together with objective criteria, for assessing the standards of datasets from each medical end-point scrutinized in this study.

**Results:** A total of 25 scientific reports comprising 10,237,230 participants for 49 meta-analyses of observational studies were selected. Among such 49 outcomes, 30 demonstrated statistical significance (*P* < 0.05). Significant associations were observed in multiple diseases, including cardiovascular/cerebrovascular, cerebral, pregnancy-related and metabolic disorders, other outcomes, and mortality.

**Conclusion:** The results showed that migraine increased the risk of 29 health outcomes, though lowered the risk of breast cancer. However, evidence quality was graded as high only for angina. The evidence quality of ischaemic stroke, stroke, MACCE, WMAs, and asthma was graded as moderate. All remaining 24 outcomes had an evidence grade of “weak.”

## Introduction

Migraine is a highly prevalent, disabling, complex primary headache-based condition, typically manifesting itself due to hyper-excitability of the central nervous system (CNS) (1). Migraine is diagnosed through multiple bouts of cranial pain and associated with a myriad of neurological symptom presentations. A migraine event is typically structured in phases: premonitory, aura, headache, postdrome, and interictal (2). Basic science studies indicate that there may be common pathways in migraine and other types of headache, such as persistent post-traumatic headache (PPTH). However, recent findings from structural and functional neuroimaging studies have attempted to describe the brain architecture of PPTH, suggesting the involvement of different networks compared to migraine (3). Migraine imposes a significant burden on patients and a great economic cost for society. It has a prevalence ranging from 2.6 to 21.7%, with a mean of 12%, depending on the population surveyed (4). Among individuals within the 30–49 year age bracket, peak migraine prevalence ranges from 11 to 20% for women and 3–8% for men, suggesting that women suffer a greater burden of migraine symptoms and disability in comparison to men (5).

In addition to causing uncomfortable symptoms including paroxysmal headaches, nausea, vomiting, photophobia, and phonophobia, migraine could exacerbate risks for incurring other adverse health outcomes. For example, earlier studies suggested that migraine patients experienced elevated risks for incurring cardiovascular diseases, including ischaemic stroke (6), hemorrhagic stroke (7), myocardial infarction (MI) (8), and angina (9). This might be explained by a number of plausible mechanisms, such as endothelial dysfunction, cerebral hypoperfusion, systemic vasculopathy, and a hypercoagulable state (10–13). Recently, emerging body of evidence from scientific literature reported the associations between migraine and other diseases, including restless leg syndrome (RLS) (14), diabetes (15), irritable bowel syndrome (IBS) (16), retinal nerve fiber layer (RNFL) thickness reduction (17), sudden sensorineural hearing loss (SSNHL) (18), major depression, and panic disorder (19). However, the published studies focused on a single health-related outcome.

Consequently, this umbrella-review study was performed for providing a detailed assessment of previously published reviews/meta-analyses that focused on the interplay between migraine and multiple heath end points. We also assessed the breadth and validity of these associations. This work suggests that migraine has a major adverse impact on human health, and will help to raise awareness of migraine and improve the motivation to treat it.

## Materials and Methods

The umbrella review was conducted in line with the PRISMA (Preferred Reporting Items for Systematic Reviews and Meta-Analyses) regulations (20), following a protocol registered with PROSPERO in advance (CRD42021273782).

### Search Strategy

The PubMed, Medline, Embase, and Web of Science were scrutinized (from repository inception date until August 2021) using “migraine” OR “headache” AND “meta-analysis” OR “systematic review” as search-terms. Furthermore, the references section for each selected article was manually scrutinized to identify potential missing meta-analyses from the initial search.

### Study Selection

Two authors (WQ and GJ) independently searched the titles and abstracts of eligible articles, followed by full text examination. All differences were discussed and resolved by consensus. Any disagreements that could not be resolved through consensus were arbitrated by a third reviewer (LZ). Articles that met the following criteria were included:

1. Meta-analyses of observational studies that evaluated the associations of migraine with any health outcomes in humans,
2. The summary effect size, with 95% confidence intervals (CIs), were available.

Whenever a single meta-analysis of multiple health outcomes was performed in one article, each outcome was included separately. Whenever multiple meta-analyses reported an identical health outcome, the meta-analysis review publication containing the highest amount of studies was selected. Systematic reviews without meta-analyses were excluded. Additionally, articles with unavailable full text were excluded. Articles discussing the increased risk of migraines from other diseases were also excluded.

### Data Extraction

WQ and GJ independently collected data using a pre-designed table containing the following parameters: outcomes, first-author and publication year, study quantity and study design, total participant quantity/cases, metric-type (OR, odds ratio; RR, relative risk; HR, hazard ratio; PR, prevalence ratio; MD, mean difference; SMD, standard mean difference), estimated summary effect and 95% confidence intervals, *P*-value for statistically significant level, *P*-value for *Q*-test, and *P*-value for Egger's test.

### Data Analysis

All summary estimates and 95% CIs were extracted directly from articles, the results being deemed to have statistical significance whenever *P* < 0.05, with *P* being collected through confidence interval using a reported method (21), whenever it was not listed in the article. The between-study heterogeneity was evaluated by the *I*^2^ statistic and Cochran's *Q*-test. Publication bias was evaluated by the Egger regression asymmetry test. *P* < 0.1 indicated statistically significant heterogeneity and publication bias. *I*^2^ < 25% was considered to be low heterogeneity, *I*^2^ > 75% was determined to be very high heterogeneity, with the remaining being classified as moderate-to-high heterogeneity.

### Evaluation of the Quality and Grading of Evidence

AMSTAR2 (22) was applied for assessing standards in methodology within all selected investigations, deemed as robust and validated instruments involved in evaluating standards within previous systematic reviews and meta-analyses. The platform ranks the quality of a meta-analysis as critically low, low, moderate and high, based upon 16 pre-determined parameters. Regarding robustness for epidemiologic proof from each medical end-point, significant correlations (*P* < 0.05) were rated as high, moderate, or weak proof, in line with a grading exercise which was previously adopted within multiple research niches (23–25). The above evaluation process was independently completed by WQ and GJ.

## Results

### Search Results

**Figure 1:**
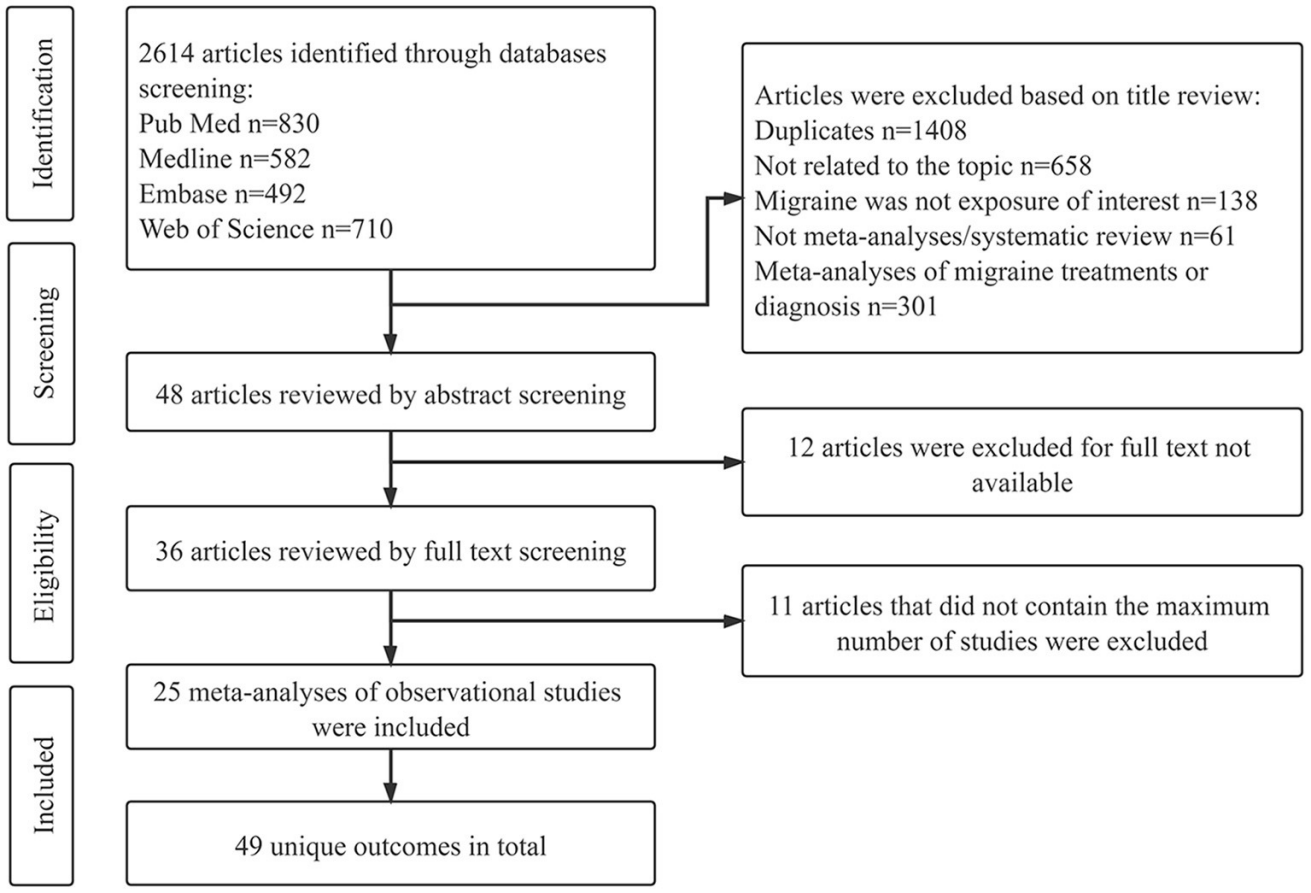
Flowchart of study selection process for umbrella review.

An in-depth flowchart for the selection protocol is illustrated in Figure 1. A total of 2,614 articles were initially identified from the four databases. 1,386 articles remained following duplicate removal, and 1,206 articles were removed from this study following scrutiny of publication title/abstract. Regarding the remaining 36 articles with full-text available, 11 were further excluded since such publications reported identical outcomes with other articles. Finally, 25 meta-analyses of observational studies, having 49 separate medical end-points were selected for this study.

**Figure 2:**
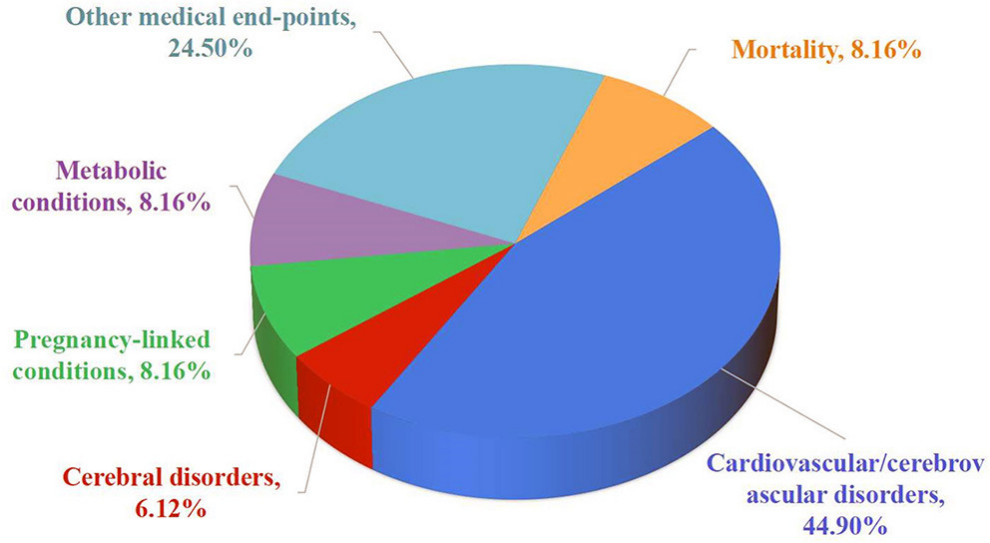
Map of outcomes related to migraine.

All 25 articles were published between 2004 and 2021. The median quantity of meta-analyses investigation including observational investigations for each medical end-point was 12 (ranged 2–30). The median participant quantity was 313,908 (ranged 330–3,945,421), while the median case quantity was 1,793 (ranged 252–383,187) (Table 1). A vast array of medical end-points were listed: cardiovascular/cerebrovascular disorders (*n* = 22), imaging abnormalities (*n* = 3), pregnancy-linked conditions (*n* = 4), metabolic conditions (*n* = 4), other medical conditions (*n* = 12), and mortality (*n* = 4) (Figure 2). From all 49 medical end-points, 30 reported effects had statistical significance (*P* < 0.05).

**Table 1:** Description of 49 meta-analyses of migraine and prevalence or incidence of diseases included in umbrella review.

**Outcomes**	**References**	**Number of studies**	**Number of participants**	**Number of cases**	**Type of metric**	**Relative risk** **(95% CI)**	**P value ^**⋆**^**	**P value ^**#**^**	**I^2^ (%)**	**P value^**※**^**	**Whether exist publication bias**
**Cardiovascular/cerebrovascular disorders**											
Ischemic stroke	Spector et al. (26)	8 cohort studies, 13 case-control studies	622,381	1,626	OR	2.04 (1.72–2.43)	<0.001	<0.001	63.5	0.66^&^	No
Hemorrhagic stroke	Sacco et al. (27)	4 cohort studies, 4 case-control studies	316,989	91,914	OR	1.48 (1.16–1.88)	0.002	0.031	54.7	0.512	No
Stroke	Mahmoud et al. (8)	7 cohort studies, 6 case-control studies	1,033,338	383,187	HR	1.42 (1.25–1.61)	<0.001	<0.001	71.6	0.66	No
Major adverse cardiovascular and cerebrovascular events (MACCE)	Mahmoud et al. (8)	3 cohort studies, 4 case-control studies	163,482	24,329	HR	1.42 (1.26–1.60)	<0.001	<0.001	40	0.87	No
Angina	Sacco et al. (9)	4 cohort studies, 1 cross-sectional study	195,905	20,443	RR	1.29 (1.17–1.43)	<0.001	0.337	12.1	0.286	No
Myocardial infarction (MI)	Sacco et al. (9)	5 cohort studies, 1 case-control study, 1 cross-sectional study	543,810	211,589	RR	1.33 (1.08–1.64)	0.007	<0.001	78.1	0.286	No
Ischemic heart disease (IHD)	Sacco et al. (9)	3 cohort studies	75,097	19,984	RR	1.48 (0.94–2.33)	0.091	<0.001	92.6	0.286	No
Coronary revascularization	Sacco et al. (9)	3 cohort studies	48,829	6,794	RR	1.11 (0.87–1.40)	0.404	0.069	62.6	0.286	No
Cervical artery dissection (CAD)	Rist et al. (28)	5 case-control studies	1,315	630	OR	2.06 (1.33–3.19)	0.001	0.061	55.5	0.14	No
Carotid artery intima-media thickness (CIMT)	Wang et al. (29)	7 case-control studies	555	279	SMD	0.84 (0.22, 1.45)	0.008	<0.001	62.39	NA	NA
Mean blood flow velocity (MBFV) in the anterior circulation	Dzator et al. (30)	30 case-control studies	4,410	2,357	SMD	0.14 (0.05, 0.23)	0.003	<0.001	47	NA	NA
Mean blood flow velocity (MBFV) in the posterior circulation	Dzator et al. (30)	18 case-control studies	3,145	1,855	SMD	0.20 (0.05, 0.34)	0.007	<0.001	68	NA	NA
Pulsatility index (PI) in the anterior circulation	Dzator et al. (30)	12 case-control studies	1,406	656	SMD	−0.02 (−0.16, 0.13)	0.83	0.05	36	NA	NA
Pulsatility index (PI) in the posterior circulation	Dzator et al. (30)	5 case-control studies	858	336	SMD	0.23 (0.05, 0.42)	0.01	0.08	38	NA	NA
Cerebrovascular responsiveness (CVR) to hypercapnia in the anterior circulation	Dzator et al. (30)	26 case-control studies	2,103	1,166	SMD	0.11 (−0.13, 0.35)	0.37	<0.001	85	NA	NA
Cerebrovascular responsiveness (CVR) to hypercapnia in the posterior circulation	Dzator et al. (30)	11 case-control studies	1,685	991	SMD	−0.34 (−0.67, −0.01)	0.04	<0.001	89	NA	NA
Cerebrovascular responsiveness (CVR) to hypocapnia in the anterior circulation	Dzator et al. (30)	8 case-control studies	352	157	SMD	0.01 (−0.43, 0.46)	0.95	<0.001	74	NA	NA
Neurovascular coupling during photic stimulation in the posterior circulation	Dzator et al. (30)	8 case-control studies	372	220	SMD	0.20 (−0.15, 0.55)	0.26	0.03	59	NA	NA
Cerebral autoregulation assessed by gain	Dzator et al. (30)	6 case-control studies	NA	NA	SMD	−0.21 (−0.43, 0.01)	0.06	NA	NA	NA	NA
Cerebral autoregulation assessed by phase	Dzator et al. (30)	6 case-control studies	NA	NA	SMD	0.13 (−0.11, 0.36)	0.29	NA	NA	NA	NA
Cerebral autoregulation assessed by Mx	Dzator et al. (30)	6 case-control studies	NA	NA	SMD	0.05 (−0.34, 0.44)	0.8	NA	NA	NA	NA
Cerebral autoregulation assessed by Dx	Dzator et al. (30)	6 case-control studies	NA	NA	SMD	0.29 (−0.08, 0.66)	0.12	NA	NA	NA	NA
**Imaging abnormalities**											
White matter abnormalities (WMAs)	Swartz and Kern (31)	7 case-control studies	629	312	OR	3.90 (2.26–6.72)	<0.001	0.66	34	0.209^&^	No
Infarct-like lesions (ILLs)	Bashir et al. (32)	2 case-control studies	3,905	522	OR	1.07 (0.87–1.33)	0.543	0.23	30.7	NA	NA
Retinal nerve fiber layer (RNFL) thickness	Lin et al. (33)	26 case-control studies	2,635	1,530	SMD	−0.53 (−0.75, −0.32)	<0.001	<0.001	85.5	NA	NA
**Pregnancy-linked conditions**											
Preeclampsia (PE)	Aukes et al. (34)	3 cohort studies, 6 case-control studies	73,892	6,799	OR	2.07 (1.51–2.85)	<0.001	<0.001	76	0.066	Yes
Lowbirth weight (LBW)	Aukes et al. (34)	2 cohort studies, 1 case-control study	69,031	5,888	OR	1.18 (1.03–1.34)	0.02	0.34	9	0.86	No
Preterm birth (PTB)	Aukes et al. (34)	3 cohort studies, 2 case-control studies	72,394	6,460	OR	1.23 (0.97–1.55)	0.09	0.04	61	0.337	No
Gestational age (SGA)	Aukes et al. (34)	2 cohort studies	30,151	5,175	OR	1.06 (0.98–1.15)	0.14	0.47	0	NA	NA
**Metabolic conditions**											
Low-density lipoprotein cholesterol (LDL-C)	Liampas et al. (35)	11 case–control studies, 1 cross-sectional study	2,585	1,370	MD	10.44 (1.64, 19.23)	0.02	<0.001	91	NA	NA
High-density lipoprotein cholesterol (HDL-C)	Liampas et al. (35)	14 case–control studies	2,816	1,488	MD	−0.37 (−2.21, 1.47)	0.69	<0.001	70	NA	NA
Total cholesterol (TC)	Liampas et al. (35)	13 case–control studies, 1 cross-sectional study	2,538	1,325	MD	10.56 (1.80, 19.31)	0.02	<0.001	85	NA	NA
Triglycerides(TG)	Liampas et al. (35)	15 case–control studies	2,788	1,526	MD	11.80 (3.62, 19.98)	0.005	<0.001	67	NA	NA
**Other medical conditions**											
Phosphene	Brigo et al. (36)	10 observational studies	330	252	OR	3.57 (1.16–10.94)	0.03	0.01	60	0.109^&^	No
Restless legs syndrome (RLS)	Wang et al. (37)	11 case–control studies	6,484	4,425	OR	3.77 (2.73–5.21)	<0.001	0.029	50.1	0.07	Yes
Epilepsy	Keezer et al. (38)	6 cohort studies	3,945,421	NA	PR	1.79 (1.43–2.25)	<0.001	<0.001	80.8	NA	NA
Breast cancer	Wu et al. (39)	3 cohort studies, 4 case-control studies	162,954	17,776	RR	0.78 (0.66–0.92)	0.003	<0.001	91.2	0.051	No
Infant colic	Zhang et al. (40)	3 cohort studies, 4 case-control studies	2,935	606	OR	2.51 (1.32–4.77)	0.005	<0.001	86	0.597^&^	No
Suicidal ideation	Friedman et al. (41)	5 cross-sectional studies	148,977	NA	OR	2.49 (2.34–2.65)	<0.001	NA	NA	0.385	No
Sudden sensorineural hearing loss (SSNHL)	Mohammadi et al. (18)	3 cohort studies	282,250	56,450	HR	1.84 (1.11–2.57)	<0.001	0.31	76.8	NA	NA
Asthma	Wang et al. (42)	3 case–control studies, 4 cross-sectional studies	395,584	156,530	OR	1.54 (1.34–1.77)	<0.001	<0.001	93	0.531	No
Depression	Amiri et al. (43)	4 cohort studies, 12 cross-sectional studies	257,077	NA	OR	1.95 (1.61–2.35)	<0.001	<0.001	92.2	0.882	No
Primary open angle glaucoma (POAG)	Xu et al. (44)	2 cohort studies, 9 case-control studies	467,008	NA	RR	1.24 (1.12–1.37)	<0.001	0.071	41.7	0.272	No
Left-handedness	Biehl et al. (45)	5 case–control studies	5,436	1,960	OR	0.93 (0.69–1.25)	0.645	NA	NA	NA	NA
attention-deficit/hyperactivity disorder (ADHD)	Salem et al. (46)	1 cohort study, 2 case-control studies, 5 cross-sectional studies	21,431	NA	OR	1.32 (1.02–1.72)	0.036	NA	NA	NA	NA
**Mortality**											
All-cause mortality	Schürks et al. (47)	5 cohort studies	424,166	NA	RR	0.90 (0.71–1.16)	0.408	<0.001	92.8	0.57	No
Cardiovascular (CVD) mortality	Schürks et al. (47)	6 cohort studies	449,074	NA	RR	1.09 (0.89–1.32)	0.398	0.02	61.4	0.54	No
Coronary heart disease (CHD) mortality	Schürks et al. (47)	3 cohort studies	60,252	NA	RR	0.95 (0.57–1.60)	0.856	0.06	64.2	0.49	No
Cardiovascular and cerebrovascular mortality	Mahmoud et al. (8)	4 cohort studies, 2 case-control studies	328,455	203,669	HR	0.93 (0.78–1.10)	0.416	0.38	91	0.81	No

*NA, not available*;

⋆ *P-value of significance level*;

# *P-value of Q test*;

※ *P-value for Egger's test*;

& *The result was reanalyzed*.

### Cardiovascular/Cerebrovascular Disorders

The adverse effects of migraine on cardiovascular and cerebrovascular diseases are well-established. Migraine patient cohorts experienced elevated risk of ischaemic stroke (26), haemorrhagic stroke (27), stroke (8), major adverse cardiovascular and cerebrovascular events (MACCE) (8), angina (9), MI (9), and cervical artery dissection (CAD) (28). Such patient cohorts also experienced increased carotid artery intima-media thickness (CIMT), indicating links between atherosclerosis and migraine (29). The scrutinized meta-analyses also revealed that migraine-sufferers possibly have an increased pulsatility index (PI) and reduced cerebrovascular responsiveness (CVR) to posterior circulatory hypercapnia (30). Other findings included an elevated resting mean blood flow velocity (MBFV) within both anterior-and posterior-circulatory migraine sufferers (30). Changes in these indicators confirm that migraine sufferers experience altered cerebrovascular faculties. There was no obvious significant association of migraine with ischaemic heart disease (IHD) and coronary revascularization (9). In addition, none of the variations (between migraineurs and controls) within the following parameters were statistically significant: anterior-circulatory PI variations; CVR to anterior-circulatory hypercapnia/hypocapnia; neurovascular coupling during photic stimulation within posterior circulation; gain-evaluated cerebral autoregulation, phase-evaluated cerebral autoregulation; Mx-evaluated cerebral autoregulation; Dx-evaluated cerebral autoregulation (30).

### Imaging Abnormalities

Migraine was related to an increased risk of white-matter abnormalities (WMAs) on magnetic resonance images (31). Compared to healthy controls, migraineurs demonstrated RNFL hypotrophy (33). However, the meta-analysis of infarct-like lesions (ILLs) on magnetic resonance images showed no association for migraineurs, when compared to controls (32).

### Pregnancy-Linked Conditions

One systematic review and meta-analysis assessed possible associations between migraine and adverse pregnancy medical end-points (34). The results showed that migraine was significantly correlated to elevated risk of preeclampsia (PE) and low birth weight (LBW), though not preterm birth (PTB) and gestational age (SGA).

### Metabolic Conditions

One systematic review and meta-analysis investigated and quantified variations in serum lipid concentrations for both migraineurs/healthy controls (35). Higher low-density lipoprotein cholesterol (LDL-C), cholesterol (TC) and triglyceride (TG) levels were found in migraineurs. The variation in high-density lipoprotein cholesterol (HDL-C) level was not statistically significant.

### Other Medical Conditions

Except for left-handedness (45), associations were found between migraine and the increased risk of phosphene (36), RLS (37), epilepsy (38), infant colic (40), suicidal contemplations (41), SSNHL (18), asthma (42), depression (43), primary open angle glaucoma (POAG) (44), together with attention-deficit/hyperactivity disorder (ADHD) (46). One meta-analysis reported a statistically significant inverse association between migraine and total breast cancer risk (39).

### Mortality

Surprisingly, although previous studies have shown that migraine has adverse effects on multiple heath medical end-points, it was not associated with mortality from cardiovascular and cerebrovascular (8), cardiovascular (CVD) (47), coronary heart disease (CHD) (47), and all-causes (47).

### Heterogeneity

Among the included meta-analyses, 33% had very high heterogeneity, 47% had moderate-to-high heterogeneity, and 6% had low heterogeneity. However, the remaining 14% did not report any heterogeneity, and this could not be re-analyzed in this study due to raw data unavailability.

### AMSTAR 2 and Summary of Evidence

**Figure 3:**
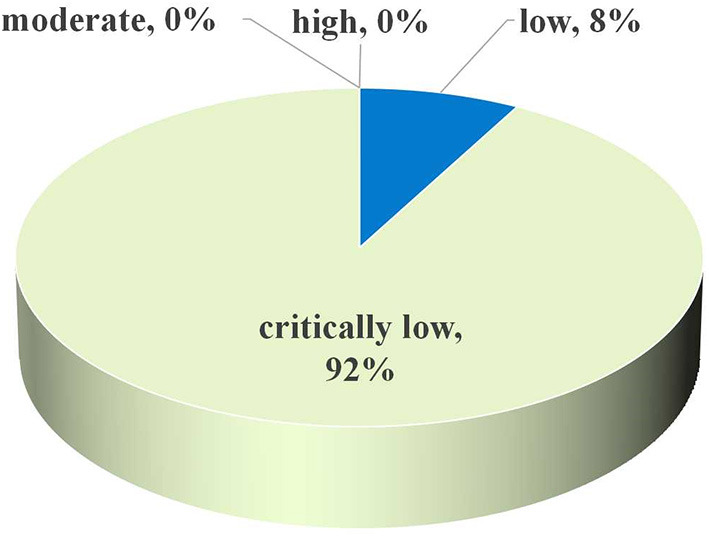
Map of results of AMSTAR 2: percentage of outcomes per outcome category for 25 meta-analyses.

**Figure 4:**
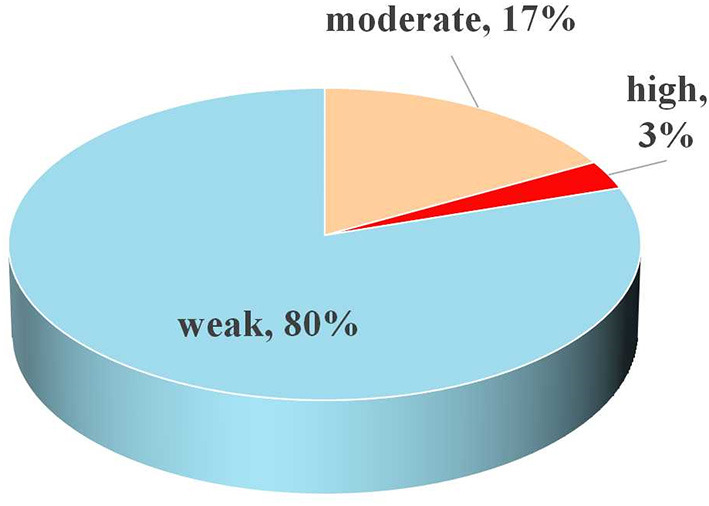
Map of results of evidence assessment: percentage of outcomes per outcome category for 30 meta-analyses.

Regarding assessment of methodological quality, only two (8%) investigations were rated as low, with the other 23 (92%) investigations rated as critically low (Figure 3). This suggested that no single investigation was deemed to carry moderate or high quality, according to AMSTAR2 standards. Following quality-of-proof for every medical end-point, ~80% were determined to be “weak” and 17% to be “moderate,” only 3% were determined to be “high” (Figure 4). Detailed information concerning AMSTAR2 and grading of evidence assessments is shown in Tables 2, 3.

**Table 2:** Detail of results for AMSTAR 2 assessing.

**References**	**AMSTAR 2 checklist**																**Overall assessment quality**
	**No. 1**	**No. 2**	**No. 3**	**No. 4**	**No. 5**	**No. 6**	**No. 7**	**No. 8**	**No. 9**	**No. 10**	**No. 11**	**No. 12**	**No. 13**	**No. 14**	**No. 15**	**No. 16**	
Spector et al. (26)	Yes	No	Yes	Yes	Yes	Yes	Partial yes	Partial yes#	Yes	No	Yes	Yes	Yes	No	Yes	Yes	Critically low
Sacco et al. (27)	Yes	No	Yes	Partial yes*	Yes	Yes	Yes	Partial yes#	Yes	No	Yes	Yes	Yes	No	Yes	No	Critically low
Mahmoud et al. (8)	Yes	Yes	Yes	Partial yes*	Yes	Yes	Yes	Partial yes#	Yes	No	Yes	Yes	Yes	Yes	Yes	Yes	Low
Sacco et al. (9)	Yes	No	Yes	Partial yes*	Yes	Yes	Yes	Partial yes#	Yes	No	Yes	Yes	Yes	No	Yes	Yes	Critically low
Rist et al. (28)	Yes	No	Yes	Partial yes*	Yes	Yes	Yes	Partial yes#	No	No	Yes	No	No	No	Yes	No	Critically low
Wang et al. (29)	Yes	No	Yes	Partial yes*	Yes	Yes	No	Partial yes#	Yes	No	Yes	Yes	Yes	No	Yes	No	Critically low
Dzator et al. (30)	Yes	No	Yes	Partial yes*	No	No	No	Partial yes#	Yes	No	Yes	Yes	Yes	No	Yes	Yes	Critically low
Swartz and Kern (31)	Yes	No	Yes	No	No	No	No	Partial yes#	No	No	Yes	No	No	No	No	No	Critically low
Bashir et al. (32)	Yes	No	Yes	No	No	No	No	Partial yes#	No	No	Yes	No	No	No	No	No	Critically low
Lin et al. (33)	Yes	Yes	Yes	Partial yes*	No	No	No	Partial yes#	Yes	No	Yes	Yes	Yes	No	No	Yes	Critically low
Aukes et al. (34)	Yes	No	Yes	Partial yes*	Yes	Yes	No	Partial yes#	Yes	No	Yes	Yes	Yes	No	No	No	Critically low
Liampas et al. (35)	Yes	No	Yes	Yes	No	No	Yes	Partial yes#	Yes	No	Yes	Yes	Yes	No	No	No	Critically low
Brigo et al. (36)	Yes	No	Yes	Yes	Yes	Yes	Yes	Partial yes#	No	No	Yes	Yes	Yes	No	Yes	Yes	Critically low
Wang et al. (37)	Yes	No	Yes	Partial yes*	Yes	Yes	No	Partial yes#	Yes	No	Yes	Yes	Yes	No	Yes	Yes	Critically low
Keezer et al. (38)	Yes	No	Yes	Yes	Yes	Yes	Yes	Partial yes#	Yes	No	Yes	Yes	Yes	No	No	Yes	Critically low
Wu et al. (39)	Yes	No	Yes	Partial yes*	Yes	Yes	No	Partial yes#	No	No	Yes	No	No	No	Yes	No	Critically low
Zhang et al. (40)	Yes	No	Yes	Partial yes*	Yes	Yes	No	Partial yes#	Yes	No	Yes	Yes	Yes	No	No	Yes	Critically low
Friedman et al. (41)	Yes	No	Yes	Partial yes*	No	No	No	Partial yes#	Yes	No	Yes	Yes	Yes	Yes	Yes	Yes	Critically low
Mohammadi et al. (18)	Yes	No	Yes	Partial yes*	Yes	Yes	No	Partial yes#	Yes	No	Yes	Yes	Yes	No	No	Yes	Critically low
Wang et al. (42)	Yes	No	Yes	Partial yes*	Yes	Yes	No	Partial yes#	Yes	No	Yes	Yes	Yes	Yes	Yes	Yes	Critically low
Amiri et al. (43)	Yes	Yes	Yes	Partial yes*	No	No	Partial yes	Partial yes#	Yes	No	Yes	Yes	Yes	Yes	Yes	Yes	Low
Xu et al. (44)	Yes	No	Yes	Partial yes*	Yes	Yes	No	Partial yes#	Yes	No	Yes	Yes	Yes	No	Yes	Yes	Critically low
Biehl et al. (45)	Yes	No	Yes	No	No	No	No	No	No	No	No	No	No	No	No	No	Critically low
Salem et al. (46)	Yes	No	Yes	No	No	No	No	No	No	No	No	No	No	No	No	Yes	Critically low
Schürks et al. (47)	Yes	No	Yes	Partial yes*	No	No	No	Partial yes#	No	No	Yes	No	No	No	Yes	Yes	Critically low

“Partial yes^*^” should meet the following requirements: (1) Search at least 2 databases related to the research question; (2) Provide keywords and/or search strategies; and (3) Explain the restrictions on literature publication, such as language restrictions. In item 8: Did the review authors describe the included studies in adequate detail? “Partial yes#” should meet the following requirements: (1) Describe the study population; (2) Describe the intervention; (3) Describe control measures; (4) Description of outcome indicators; and (5) Describe the type of study.

**Table 3:** Detail of results for evidence quality assessing.

**Outcomes**	**References**	**Precision of the estimate**		**Consistency of results**	**No evidence of small-study effects**	**Grade**
		**>1,000 disease cases**	***P* < 0.001**	**(I^2^ < 50% and Cochran *Q*-test *P* > 0.10)**	**(*P* > 0.10)**	
**Cardiovascular/cerebrovascular disorders**						
Ischemic stroke	Spector et al. (26)	Yes	Yes	No	Yes	Moderate
Hemorrhagic stroke	Sacco et al. (9)	Yes	No	No	Yes	Weak
Stroke	Mahmoud et al. (8)	Yes	Yes	No	Yes	Moderate
Major adverse cardiovascular and cerebrovascular events (MACCE)	Mahmoud et al. (8)	Yes	Yes	No	Yes	Moderate
Angina	Sacco et al. (9)	Yes	Yes	Yes	Yes	High
Myocardial infarction (MI)	Sacco et al. (9)	Yes	No	No	Yes	Weak
Cervical artery dissection (CAD)	Rist et al. (28)	No	No	No	Yes	Weak
Carotid artery intima-media thickness (CIMT)	Wang et al. (29)	No	No	No	No	Weak
Mean blood flow velocity (MBFV) in the anterior circulation	Dzator et al. (30)	Yes	No	No	No	Weak
Mean blood flow velocity (MBFV) in the posterior circulation	Dzator et al. (30)	Yes	No	No	No	Weak
Pulsatility index (PI) in the posterior circulation	Dzator et al. (30)	No	No	No	No	Weak
Cerebrovascular responsiveness (CVR) to hypercapnia in the posterior circulation	Dzator et al. (30)	No	No	No	No	Weak
**Imaging abnormalities**						
White matter abnormalities (WMAs)	Swartz and Kern (31)	No	Yes	Yes	Yes	Moderate
Retinal nerve fiber layer (RNFL) thickness	Lin et al. (33)	Yes	Yes	No	No	Weak
**Pregnancy-linked conditions**						
Preeclampsia (PE)	Aukes et al. (34)	Yes	Yes	No	No	Weak
Low birth weight (LBW)	Aukes et al. (34)	Yes	No	Yes	Yes	Weak
**Metabolic conditions**						
Low-density lipoprotein cholesterol (LDL-C)	Liampas et al. (35)	Yes	No	No	No	Weak
Total cholesterol (TC)	Liampas et al. (35)	Yes	No	No	No	Weak
Triglycerides (TG)	Liampas et al. (35)	Yes	No	No	No	Weak
**Other medical conditions**						
Phosphene	Brigo et al. (36)	No	no	no	yes	Weak
Restless legs syndrome (RLS)	Wang et al. (37)	Yes	yes	No	No	Weak
Epilepsy	Keezer et al. (38)	No	Yes	No	No	Weak
Breast cancer	Wu et al. (39)	Yes	No	No	No	Weak
Infant colic	Zhang et al. (40)	No	No	No	Yes	Weak
Suicidal ideation	Friedman et al. (41)	No	yes	No	Yes	Weak
Sudden sensorineural hearing loss (SSNHL)	Mohammadi et al. (18)	Yes	yes	No	No	Weak
Asthma	Wang et al. (42)	Yes	Yes	No	Yes	Moderate
Depression	Amiri et al. (43)	No	Yes	No	Yes	Weak
Primary open angle glaucoma (POAG)	Xu et al. (44)	No	Yes	No	Yes	Weak
attention-deficit/hyperactivity disorder (ADHD)	Salem et al. (46)	No	No	No	No	Weak

## Discussion

This umbrella review identified 49 unique health medical end-points from 25 studies. The results provided a broad overview of the current evidence of relationships between migraine and various health medical end-points, including cardiovascular/cerebrovascular disorders, cerebral disorders, pregnancy-linked conditions, metabolic conditions, other medical end-points, and mortality. Among these, 30 meta-analyses registered statistically significant results, whereby migraine was linked to reduced breast cancer risk and an increased risk of 29 other medical end-points. However, the evidence quality was graded as high only for angina. The evidence quality of ischaemic stroke, stroke, MACCE, WMAs, and asthma was graded as moderate, while the remaining 24 medical end-points had an evidence grade of “weak.”

The International Classification of Headache Diseases (ICHD) has discerned between migraine with aura (MA) and migraine without aura (MO) based on the presence/absence of spreading oligemia (48). The similarities and differences of pathophysiologic, epidemiologic, and clinical proof between migraine with/without aura were reviewed in early studies. Migraine, particularly MA, correlated with exacerbated risk for ischemic/hemorrhagic stroke events (49, 50). The sub-group investigations of ischemic stroke, hemorrhagic stroke, stroke, MI, angina, MBFV in the anterior circulation, RNFL thickness, and phosphene within this umbrella review, showed similar results. Migraine is also 2–3 × fold more prevalent in women (51). Although many studies show no difference in mean pain intensity between men and women, headache-related disability is reported more frequently in women (52–54). Results of the gender-specific subgroup analyses in this umbrella review showed that the risks of ischemic stroke, hemorrhagic stroke, MI, and angina were elevated in female migraineurs. Cohort study was not greatly influenced through recall/selection biases and was less prone to bias through reverse causality, in comparison to case-control/cross-sectional investigation (55). Correlations between migraine and disease can lead to differing results, depending upon study design. For example, there was a statistically significant inverse association between migraine breast cancer event risk. However, such an inverse relationship was recognized within case–control investigations, though not within cohort investigations. This was consistent with the results of another study (56), which was excluded from this umbrella review. Consequently, larger quantities of prospective cohort studies are required to verify such a correlation.

Migraine was associated with 30 medical end-points. However, serious heterogeneity between studies existed in most of the meta-analyses. The following factors contributed to the heterogeneity of the included meta-analyses: age, geographical area, migraines ascertainment, migraine aura status, and study design. The standards of methods implemented in all selected meta-analyses was categorized as “critically low” or “low,” mostly because of a “no” decision on the following items: a pre-recognized explicit statement/protocol, a list of excluded studies, bias risk assessment in selected studies, funding source details for the selected studies, discussion of heterogeneity observed within review results, report of potential sources for conflicts of interest. Only one medical end-point was rated as high quality-of-proof. Many studies did not report results for *I*^2^ statistic, *P*-value for Cochran's *Q*-test, and *P*-value for Egger's test, leading to a decline in evidence grade.

The authors believe this is a pioneering investigational effort to assess properly all links between migraine and multiple health/medical end-points through adoption of an umbrella review approach. The authors performed a critical appraise of the range and validity of reported relationships between migraine and diverse health/medical end-points. Notwithstanding, some limitations inevitably existed in this umbrella review. Firstly, results of individual observational investigations involving under-developed meta-analysis were beyond the scope of this review, such as the concentration of lipoprotein(a) (57) and diabetes (58). Thus, we might have missed some researches on the links between migraine and multiple health/medical end-points. Secondly, when two or more meta-analyses reported identical health/medical end-points, the report containing the most studies was selected, regardless of study design. Therefore, the results may be skewed by the influence of recall/selection bias and reverse causality. Thirdly, one study reported that migraine increased the risk of IBS, though this umbrella review did not select this study since the full-text was not available. We tried to contact the author to obtain the full text, but failed, which resulted in the loss of a very important research result. Fourthly, this umbrella review did not include publications in languages other than English. The link between migraines and health/medical end-points reported in other languages may have been overlooked. Consequently, conclusion bias of association between migraine and human health can be produced by the aforementioned situations.

In conclusion, this review provided a detailed evaluation of all available data on links between migraine and various health/medical end-points. The results showed that migraine increased the risk of 29 health/medical end-points and reduced the risk of breast cancer. Considering that evidence for most medical end-points were categorized as “moderate” and “weak,” additional high-quality prospective cohort studies are required in order to draw a firm conclusion.

## Data Availability Statement

The original contributions presented in the study are included in the article/supplementary material, further inquiries can be directed to the corresponding author.

## Author Contributions

WC: idea, design, and manuscript revision. WQ and GJ: literature search, data extraction, and analysis. LZ: manuscript writing. All authors read and approved the version of the manuscript to be published and took responsibility for appropriate content.

## Funding

This study was supported by 2021 Zunyi Science and Technology Bureau and Zunyi First People's Hospital joint science and Technology Research and development fund project [Zun Kehe HZ Word (2021) No. 233].

## Conflict of Interest

The authors declare that the research was conducted in the absence of any commercial or financial relationships that could be construed as a potential conflict of interest.

## Publisher's Note

All claims expressed in this article are solely those of the authors and do not necessarily represent those of their affiliated organizations, or those of the publisher, the editors and the reviewers. Any product that may be evaluated in this article, or claim that may be made by its manufacturer, is not guaranteed or endorsed by the publisher.

## Abbreviations

PPTH: persistent post-traumatic headache
CNS: central nervous system
MI: myocardial infarction
RLS: restless leg syndrome
IBS: irritable bowel syndrome
RNFL: retinal nerve fiber layer
SSNHL: sudden sensorineural hearing loss
PRISMA: preferred reporting items for systematic reviews and meta-analyses
CIs: confidence intervals
OR: odds ratio
RR: relative risk
HR: hazard ratio
PR: prevalence ratio
MD: mean difference
SMD: standard mean difference
MACCE: major adverse cardiovascular and cerebrovascular events
CAD: cervical artery dissection
CIMT: carotid artery intima-media thickness
PI: pulsatility index
CVR: cerebrovascular responsiveness
MBFV: mean blood flow velocity
IHD: ischaemic heart disease
WMAs: white-matter abnormalities
ILLs: infarct-like lesions
PE: preeclampsia
LBW: low birth weight
PTB: preterm birth
SGA: gestational age
LDL-C: low-density lipoprotein cholesterol
TC: cholesterol
TG: triglyceride
HDL-C: high-density lipoprotein cholesterol
POAG: primary open angle glaucoma
ADHD: attention-deficit/hyperactivity disorder
CVD: cardiovascular
CHD: coronary heart disease
ICHD: international classification of headache diseases
MA: migraine with aura
MO: migraine without aura.
